# Earth's Climate History Explains Life's Temperature Optima

**DOI:** 10.1002/ece3.70701

**Published:** 2024-12-12

**Authors:** Louis A. Schipper, Jennifer L. Reeve, Vickery L. Arcus, Terry Isson, Erica J. Prentice, Adele Williamson, Andrew D. Barnes

**Affiliations:** ^1^ Te Aka Mātuatua School of Science The University of Waikato Hamilton Aotearoa New Zealand; ^2^ Te Aka Mātuatua School of Science The University of Waikato Tauranga Aotearoa New Zealand

**Keywords:** early evolution, selection pressure, temperature optima

## Abstract

Why does the growth of most life forms exhibit a narrow range of optimal temperatures below 40°C? We hypothesize that the recently identified stable range of oceanic temperatures of ~5 to 37°C for more than two billion years of Earth history tightly constrained the evolution of prokaryotic thermal performance curves to optimal temperatures for growth to less than 40°C. We tested whether competitive mechanisms reproduced the observed upper limits of life's temperature optima using simple Lotka–Volterra models of interspecific competition between organisms with different temperature optima. Model results supported our proposition whereby organisms with temperature optima up to 37°C were most competitive. Model results were highly robust to a wide range of reasonable variations in temperature response curves of modeled species. We further propose that inheritance of prokaryotic genes and subsequent co‐evolution with microbial partners may have resulted in eukaryotes also fixing their temperature optima within this narrow temperature range. We hope this hypothesis will motivate considerable discussion and future work to advance our understanding of the remarkable consistency of the temperature dependence of life.

## Graphical abstract

We propose that the origin of the temperature optima of life results from two discrete evolutionary selection pressures that constrained the evolution of fundamental biochemistry and growth of most lifeforms to be optimized below 40°C. Our work builds on a recent paper that shows maximum oceanic temperatures remained remarkably stable and below 37°C for more than two and a half billion years. We demonstrate how these competitive mechanisms can produce the observed upper limits of life's temperature optima using simple Lotka–Volterra models of interspecific competition between organisms with different temperature optima.

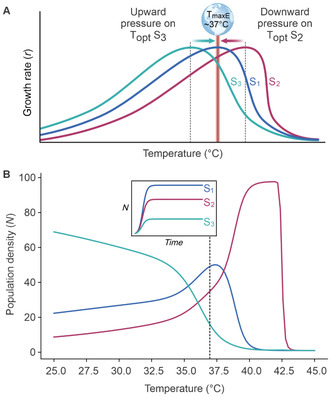

## 1 Introduction

**FIGURE 1:**
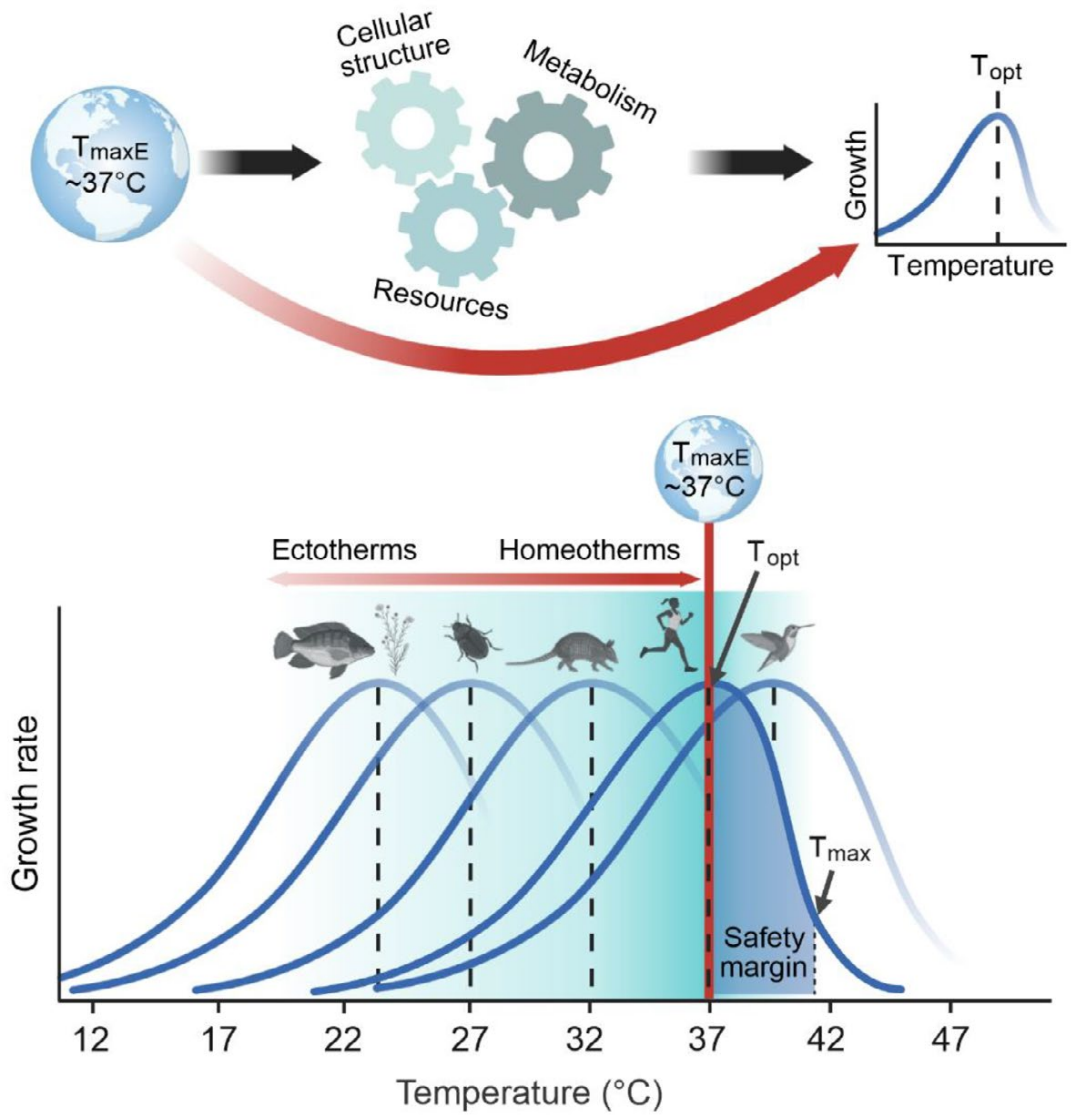
Earth's temperature history determined life's thermal optima. We propose that stable ocean temperatures with an upper limit of ~37°C since the Archean (*T*_maxE_) imposed strong selection pressures on the temperature optima of early life via thermal constraints on cell structure, metabolism, and resource acquisition. These macroevolutionary pressures likely gave rise to the observed universal upper limit of temperature optima (*T*_opt_) in modern lifeforms.

Growth and reproduction for most organisms, including microbes, plants, and animals (ectotherms and homeotherms), reach a maximum at mesophilic temperatures (< 40°C; Arroyo et al. 2022; Dell, Pawar, and Savage 2011; Gillooly et al. 2001; Sørensen et al. 2018). Above an organism's temperature optimum (*T*_opt_), there is often a rapid decline in growth rate. Below *T*_opt_, growth rate gradually declines to near zero at 0°C. This asymmetric temperature response is determined by the temperature dependence of metabolic pathways, maintenance of cellular structures, and ability to access resources (Araújo et al. 2013; Bennett et al. 2021; Dell, Pawar, and Savage 2011; Figure 1). The origin of this remarkably consistent upper bound of *T*_opt_ remains unknown but has been suggested to lie in Earth's paleo‐environmental history (Bennett et al. 2021).

Earth's surface temperature history has been a controversial topic for a few decades (e.g., Jaffrés, Shields, and Wallmann 2007). Recently, the use of a new paired mineral oxygen isotope approach revealed that stable ocean temperatures did not exceed ~37°C on average for at least the last two billion years of Earth's history (Isson and Rauzi 2024). Furthermore, independent sedimentological and geochemical evidence suggest that the preceding Archean eon was characterized by similarly temperate conditions (Isson and Rauzi 2024).

**FIGURE 2:**
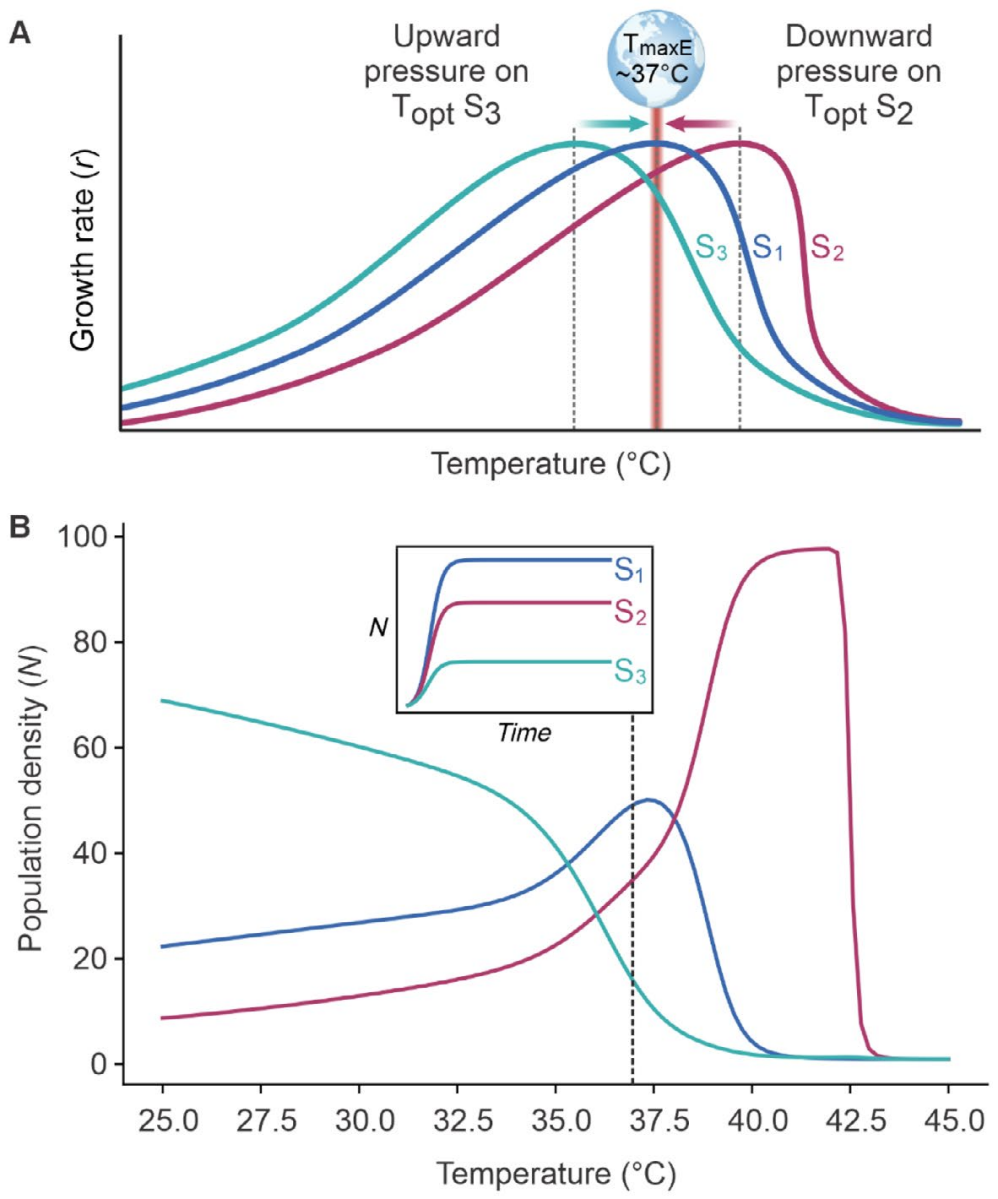
Organisms receive a competitive advantage when optimizing growth rates to local environmental temperatures. We propose that selection was driven by competitive advantages of increased growth rates for organisms with *T*_opt_ that closely match environmental temperatures due to opposing upward and downward selection pressures on organismal temperature optima (A). Simple competition models (B) suggest organisms with lower *T*_opt_ mainly face competitive constraints, whereas organisms with the highest *T*_opt_ only escape competition at warmer extremes but eventually face strong maintenance costs and death as they approach upper critical temperatures for growth (*T*_max_). Inset in (B) shows the competitive dynamics of three model species at an environmental temperature of 37°C.

Here, we consider how this consistent temperature window constrained the evolution of *T*_opt_ for growth in prokaryotes and the subsequent emergence of eukaryotes. We propose that the average global maximum temperature of the environment (*T*_maxE_) imposed strong selection pressures on the upper bound of growth *T*_opt_ in early prokaryote communities via competitive dynamics (Comeault and Matute 2021; Hillebrand 2011; Figure 1). This upper bound was constrained through two mechanistically distinct pressures that select against species with growth *T*_opt_ above or below maximal environmental temperatures (Figure 2A). For warmer periods in Earth history (relative to the modern climate), evidence for a reduced equator‐to‐pole temperature gradient (Evans et al. 2018) suggests that life on Earth was subject to a more uniform climate with fewer colder and hotter refugia. This would have imposed a more globally homogeneous thermal selection pressure on the *T*_opt_ of life forms toward ~37°C.

### 1.1 Conceptual Framework

We considered competition between populations of three species at the proposed *T*_maxE_ of 37°C, each with a different *T*_opt_ (Figure 2A): *T*_opt_ of 37°C (species 1; S_1_), > 37°C (species 2; S_2_), and < 37°C (species 3; S_3_). At temperatures below *T*_maxE_, S_3_ holds a competitive advantage over organisms with a higher *T*_opt_ (e.g., S_1_ or S_2_). However, when the environmental temperature exceeds *T*_opt_ of S_3_, they face large competitive penalties as individuals must invest energy into maintenance rather than growth unless they can retreat to colder refugia. Simply, this pressure will select for organisms with higher *T*_opt_ approaching *T*_maxE_. The inverse is true for S_2_ with a *T*_opt_ above *T*_maxE_ (Figure 2A). These organisms can outcompete S_1_ and S_3_ but only at temperatures above *T*_maxE_ rarely observed during Earth's multi‐billion‐year history. This implies a persistent downward selection pressure on the *T*_opt_ of organismal growth. These two selection pressures do not have equal strength, in that the downward pressure exerts a greater constraint on the upper limit of *T*_opt_ (Bennett et al. 2021; MacLean et al. 2019). This is because organisms with a *T*_opt_ higher than the *T*_maxE_ (such as S_2_) would have been poor competitors spanning the majority of Earth's history during which life evolved and diversified. In contrast, organisms with a *T*_opt_ lower than *T*_maxE_ (such as S_3_; Figure 2A) are more likely to experience temperatures that allow for maximal growth. In this framework, organisms with lower *T*_opt_ are allowed to be competitive across a broader and lower range of temperatures.

## 2 Results and Discussion

We test this conceptual framework using a simple Lotka–Volterra model of interspecific competition for three organisms with different *T*_opt_ (see methods). By running the competition model across a range of temperatures, we demonstrate that organisms with lower *T*_opt_ (such as S_3_) dominate at lower temperatures at the expense of any competitive advantage at higher temperatures (Figure 2B). Conversely, organisms with higher *T*_opt_ (such as S_2_) have a competitive disadvantage at lower temperatures in exchange for a major competitive advantage at higher temperatures. Although the range of temperatures at which an organism with an intermediate *T*_opt_ (such as S_1_) outcompetes all other organisms is narrow, it can survive and maintain reasonable population densities across the majority of the environmentally relevant temperature range (Figure 2B). Despite its simplicity, this competition model was highly robust to extensive variations in the thermal performance curves among competing organisms, including thermal sensitivities, temperature optima, and growth curve skewness (see appendices). These various scenarios were based on known interspecific variation in temperature performance curves within and among different lifeforms (Dell, Pawar, and Savage 2011; Kordas, Harley, and O'Connor 2011; Sørensen et al. 2018). It is important to note that many other temperature‐dependent biological rates are not included in the classic Lotka–Volterra competition model (i.e., other than growth) that could alter the outcome of competitive interactions. For example, temperature positively affects movement speed, consumer–resource encounter rates, and consumption rates, all of which will further amplify the thermal dependence of interspecific competition and adaptation to optimal environmental temperatures (Dell, Pawar, and Savage 2014). Nevertheless, more complex models that incorporate additional temperature‐dependent biological rates should produce equivalent conclusions, due to similar asymptotic thermal performance curves around a temperature optimum (Dell, Pawar, and Savage 2014) that are comparable to growth curves. On protracted timescales, these two selection pressures (Figure 2B) result in the optimal temperature for growth at and below *T*_maxE_ (Dell, Pawar, and Savage 2011; Sørensen et al. 2018). Our results demonstrate that temperature optima, alongside minimum and maximum temperature tolerances of organisms, are critical in determining the ecological and evolutionary consequences of shifting environmental temperatures.

This proposed framework does not preclude the evolution of thermophiles and psychrophiles (Engqvist 2018) that maintain lifetime reproductive success at more extreme temperatures. Critically, we note that enzyme *T*_opt_ values are significantly higher than microbial growth temperatures for psychrophiles (Stark et al. 2022) and lower for thermophiles. This suggests that, to cope with the disconnect between enzyme performance and environmental temperature, extremophiles have evolved other metabolic adaptations such as heat shock proteins in thermophiles (Ezemaduka et al. 2014) that incur high maintenance costs, which make them less competitive in mesophilic environments. Despite these exceptions, the thermal performance of life appears to gravitate toward an optimum at 37°C because of the positive scaling of biological rates with temperature (Dell, Pawar, and Savage 2011).

The temperature optima of early unicellular life may also be responsible for the remarkably consistent upper bound of thermal optima of modern organisms (Dell, Pawar, and Savage 2011; Sørensen et al. 2018). The transfer of thermal constraints to more complex multicellular lifeforms could have arisen via multiple pathways. One possibility is that the consistent evolution of *T*_opt_ for growth in early prokaryotes resulted in a biochemical system optimized to *T*_maxE_ so complex that it was inescapable under subsequent changes in environmental temperature. Core components of metabolism are thought to be present in the Last Universal Common Ancestor (LUCA) including the ribosome (protein synthesis), the TCA cycle and gluconeogenesis which link carbon fixing to nucleotide biosynthesis (Petrov et al. 2015; Smith and Morowitz 2004). Temperature constraints on these core processes may be fixed early in evolution and thus their expression today is a relic of early evolutionary innovations (Petrov et al. 2015; Smith and Morowitz 2004). Thus, the temperature optimization of early unicellular organisms could have led to the preordained thermal properties of metabolism in evolving lifeforms such as metazoans, plants, fungi, and algae. The constrained temperature response of inherited biochemistry would be further re‐enforced during the co‐evolution of the thermal performance of hosts and their microbiomes (Zilber‐Rosenberg and Rosenberg 2008). This suggests most eukaryote hosts and their prokaryote partners faced the same selection pressures to optimize growth below ~37°C for mutual benefit.

If temperature‐dependent competition has shaped the evolution of organisms because of stable temperatures below ~37°C for more than two billion years, this raises a multitude of intriguing questions. As metabolism regulates processes at the cellular level and in turn global biogeochemical cycles, how has Earth's temperature history shaped biodiversity and ecosystem processes from micro‐ to macro‐scales and how have these processes created feedback to constrain changes in global temperatures through time? Further, does the existing *T*_opt_ of life act as a hard constraint for ongoing adaptation to increasing global temperatures? Exploring these questions can advance our understanding of the past, present, and future variability of Earth's biodiversity.

## 3 Methods

We modeled the influence of mean environmental temperature on interspecific competition among three hypothetical species with different *T*_opt_ using a simple Lotka–Volterra competition model (Reuman, Holt, and Yvon‐Durocher 2014) whereby the intrinsic growth rates, *r*, of competing species were temperature dependent:

$$\frac{dN_{1}}{\mathrm{dt}}=r_{1}\left(T\right)N_{1}\left[1-\frac{\sum_{i=1}^{n}N_{i}}{K_{1}}\right]\left(1\right)$$

$$\begin{array}{c} \frac{dN_{2}}{\mathrm{dt}}=r_{2}\left(T\right)N_{2}\left[1-\frac{\sum_{i=1}^{n}N_{i}}{K_{2}}\right]\left(2\right) \end{array}$$

$$\begin{array}{c} \frac{dN_{3}}{\mathrm{dt}}=r_{3}\left(T\right)N_{3}\left[1-\frac{\sum_{i=1}^{n}N_{i}}{K_{3}}\right]\left(3\right) \end{array}$$

**FIGURE A1:**
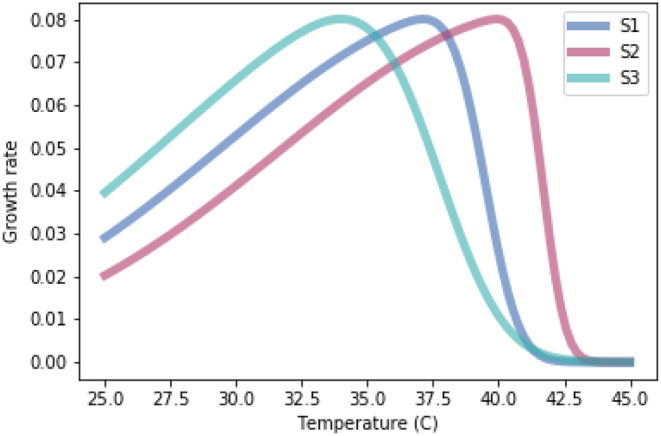
Growth rate–temperature relationships used for the competition model in Figure 1.3.

**FIGURE A2:**
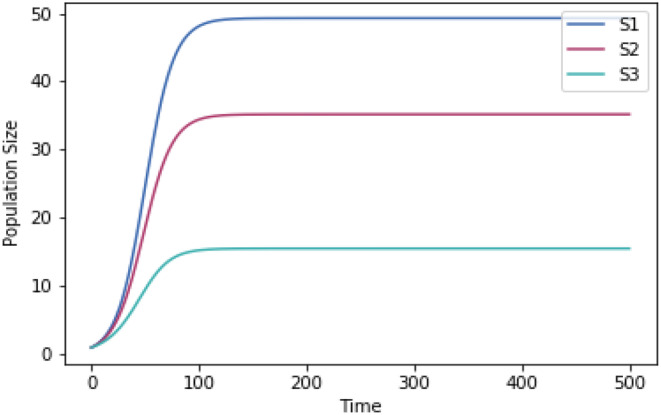
Competition of all three species at 37°C.

In this model, *N*_*i*_ is the density of competitor *i* and *K*_*i*_ is the carrying capacity of species *i*. In our initial model, temperature–growth curves for maximal intrinsic growth rates, *r*, were varied among species, with S_1_ having intermediate skewedness and *T*_opt_, S_2_ having high skewedness and *T*_opt_, and S_3_ having low skewedness and *T*_opt_ (Table A1) to mimic potential scenarios of varied temperature–growth curves (Kordas, Harley, and O'Connor 2011). The temperature–growth curves were normalized to have the same temperature‐dependent maximum growth rate for each species (Figure A1). At each temperature tested (spanning a continuous range from 25°C to 45°C), three‐species interspecific competition models were run to steady state to determine outcomes in the relative population sizes for each species (Figure A2).

**FIGURE A3:**
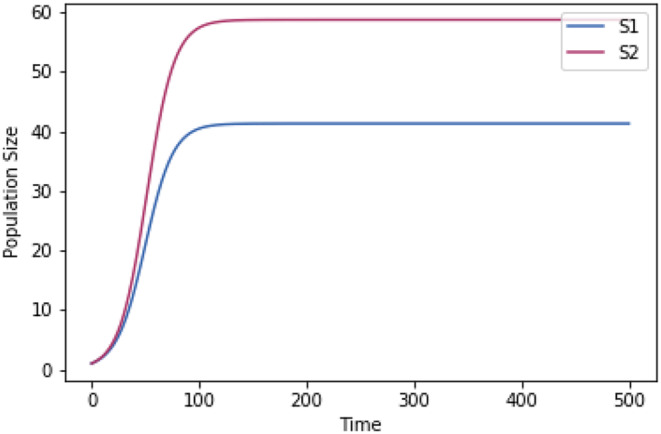
Competition of S1 and S2 at 37°C.

**FIGURE A4:**
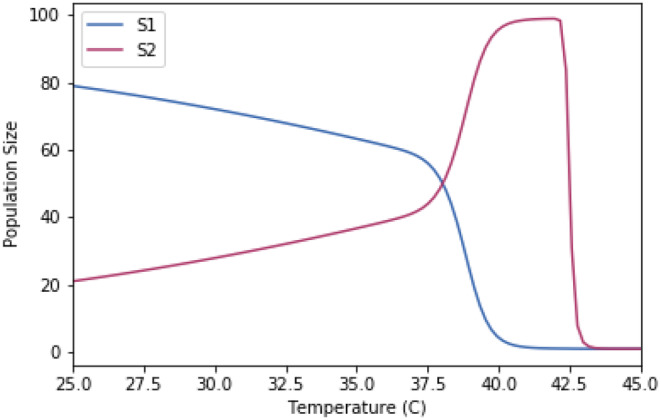
Competition of S1 and S2 across 25.0°C–45.0°C.

**FIGURE A5:**
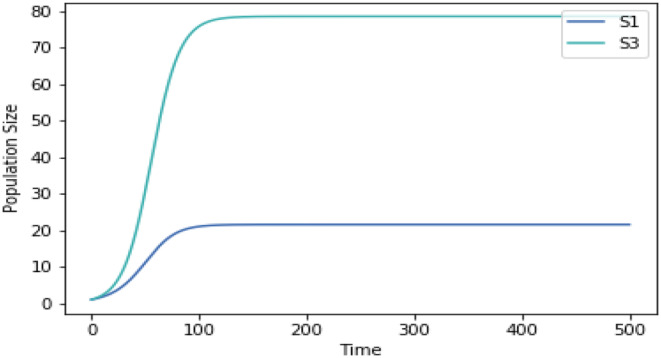
Competition of S1 and S3 at 37°C.

**FIGURE A6:**
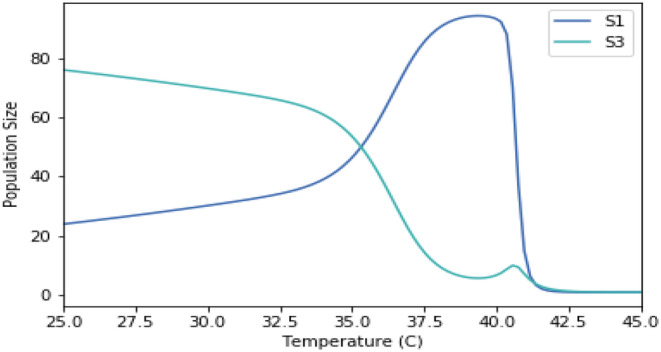
Competition of S1 and S3 across 25.0°C–45.0°C.

**FIGURE A7:**
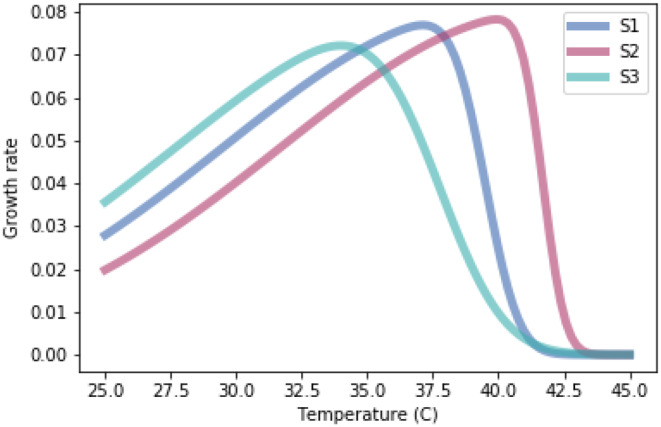
Growth rate–temperature relationships without normalization.

**FIGURE A8:**
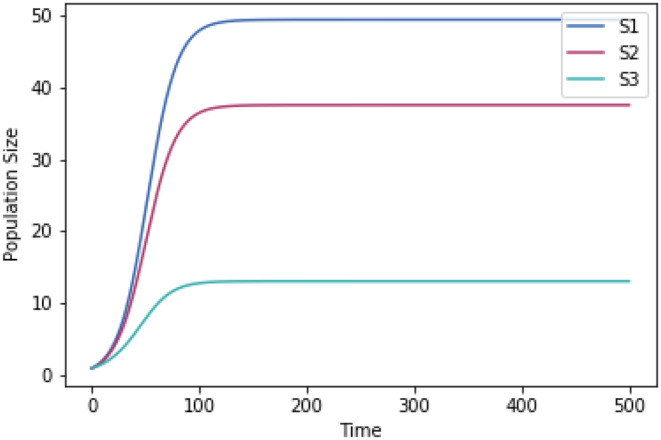
Competition of species at 37°C without normalization of the growth rate–temperature relationships.

**FIGURE A9:**
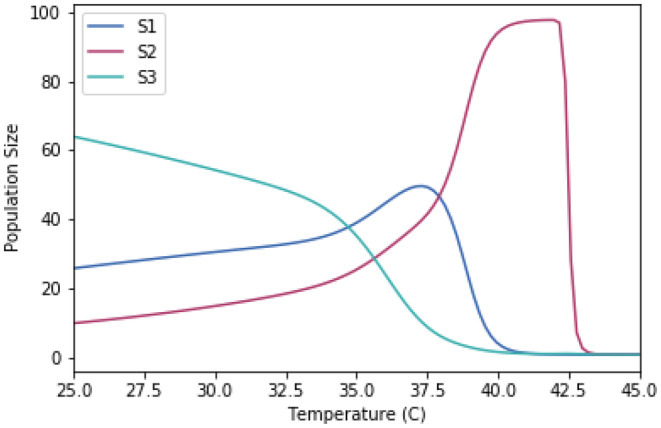
Competition of species across 25.0°C–45.0°C without normalization of the growth rate‐temperature relationships.

**FIGURE A10:**
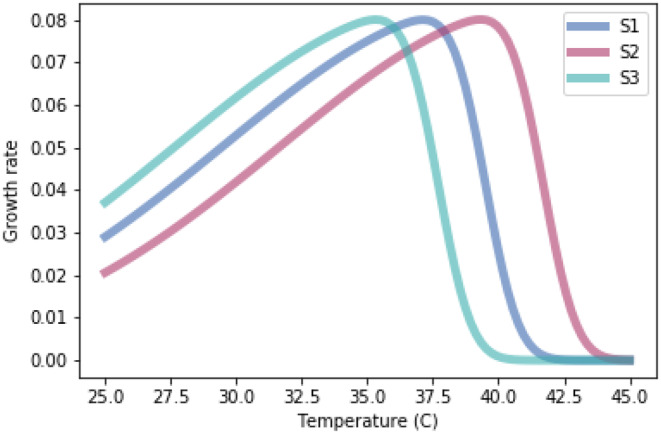
Growth rate–temperature relationships without differences in skew.

**FIGURE A11:**
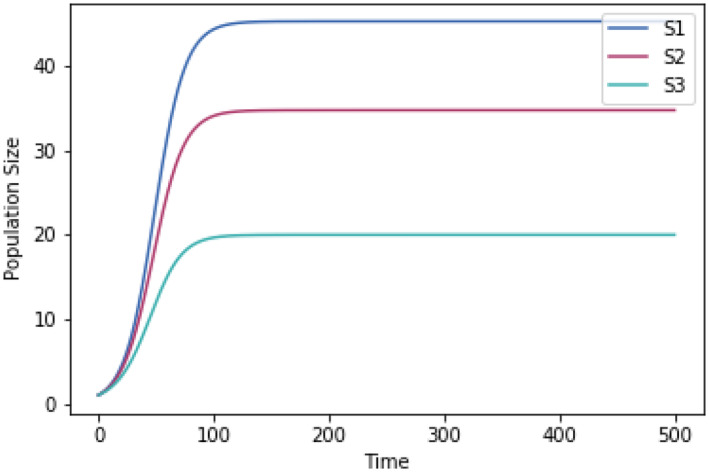
Competition of species at 37°C without differences in the skew of the growth rate–temperature relationships.

**FIGURE A12:**
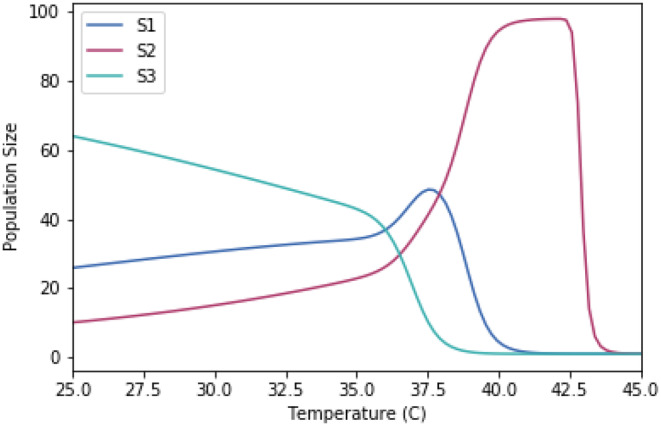
Competition of species across 25.0°C–45.0°C without differences in skew of the growth rate–temperature relationships.

**FIGURE A13:**
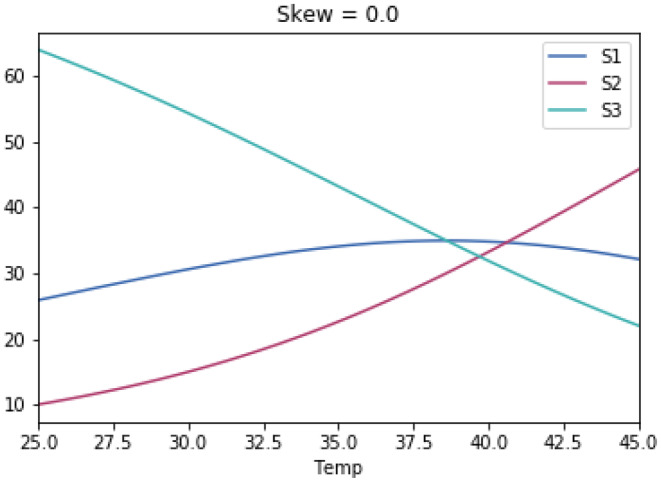
Competition of species across 25.0°C–45.0°C with skewedness of 0.0 for all of the growth rate–temperature relationships.

**FIGURE A14:**
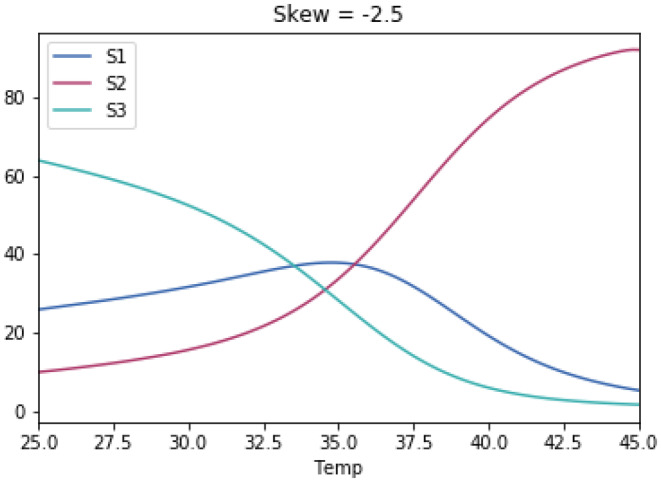
Competition of species across 25.0°C–45.0°C with skewedness of −2.5 for all of the growth rate–temperature relationships.

**FIGURE A15:**
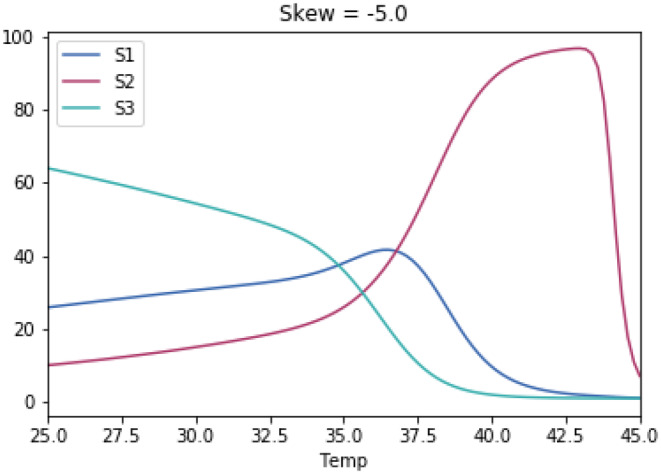
Competition of species across 25.0°C–45.0°C with skewedness of −5.0 for all of the growth rate–temperature relationships.

**FIGURE A16:**
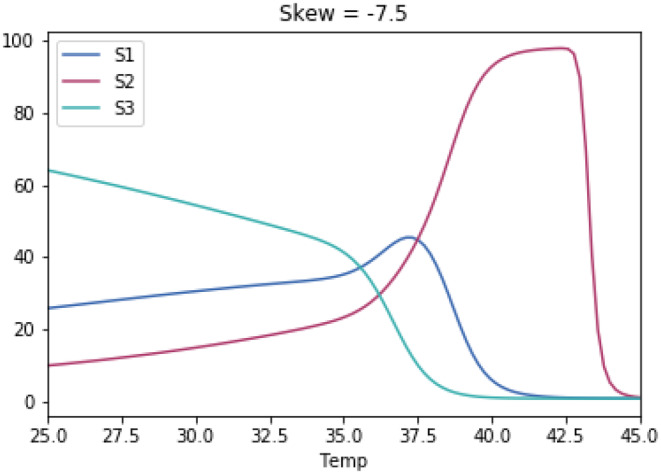
Competition of species across 25.0°C–45.0°C with skewedness of −7.5 for all of the growth rate–temperature relationships.

**FIGURE A17:**
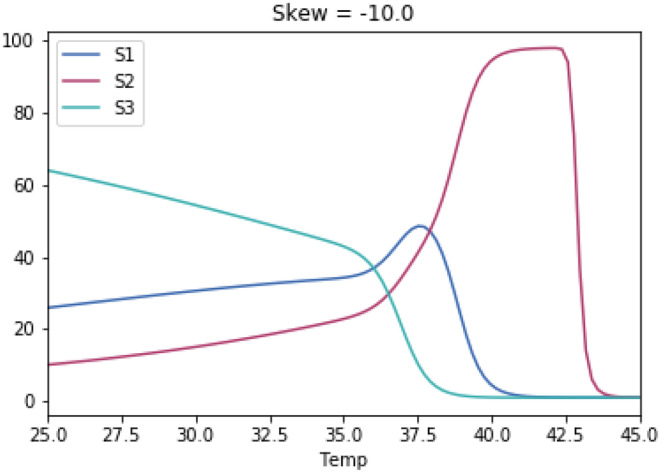
Competition of species across 25.0°C–45.0°C with skewedness of −10.0 for all of the growth rate–temperature relationships.

**FIGURE A18:**
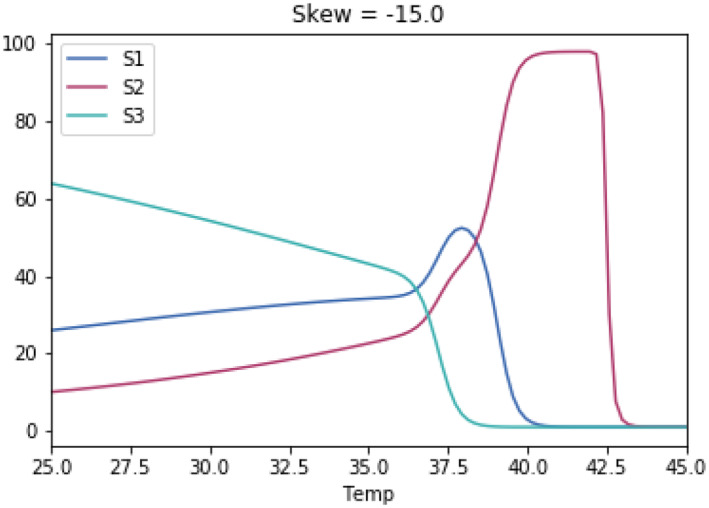
Competition of species across 25.0°C–45.0°C with skewedness of −15.0 for all of the growth rate–temperature relationships.

**FIGURE A19:**
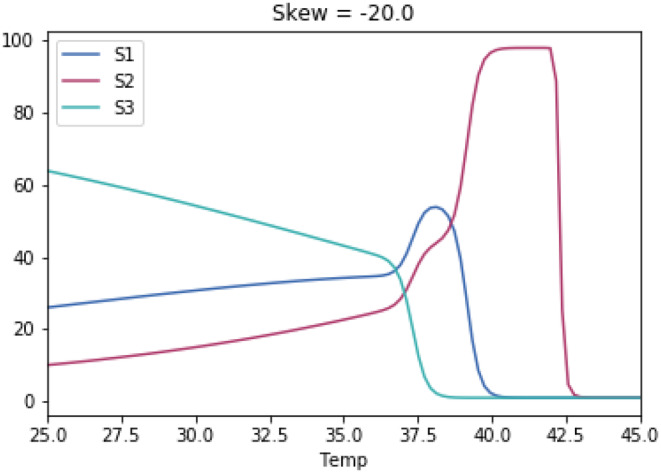
Competition of species across 25.0°C–45.0°C with skewedness of −20.0 for all of the growth rate–temperature relationships.

**FIGURE A20:**
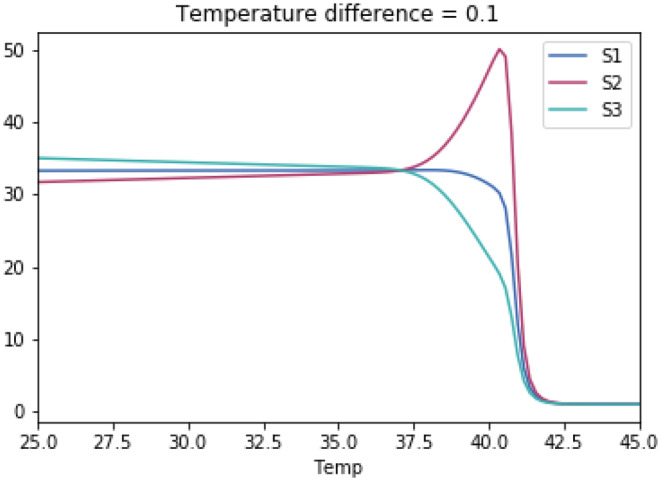
Competition of species across 25.0°C–45.0°C with differences in the temperature optimum of 0.1°C.

**FIGURE A21:**
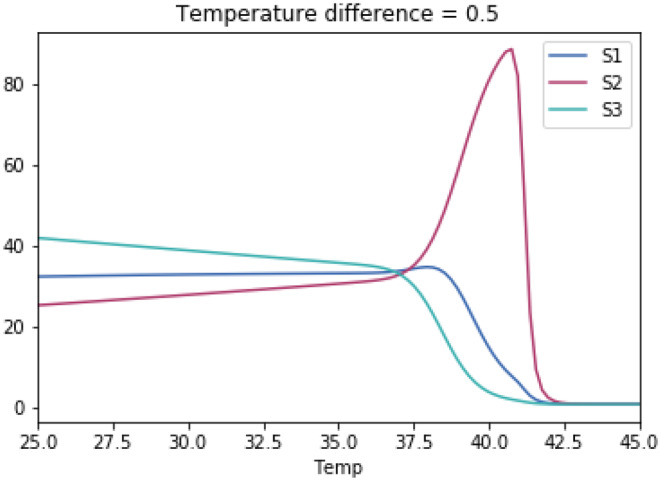
Competition of species across 25.0°C–45.0°C with differences in the temperature optimum of 0.5°C.

**FIGURE A22:**
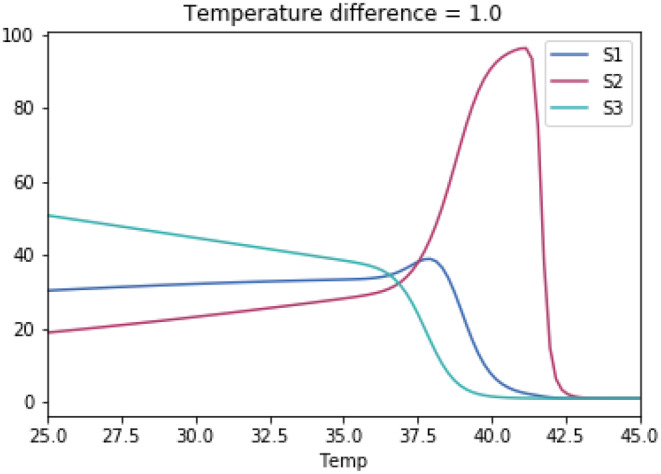
Competition of species across 25.0°C–45.0°C with differences in the temperature optimum of 1.0°C.

**FIGURE A23:**
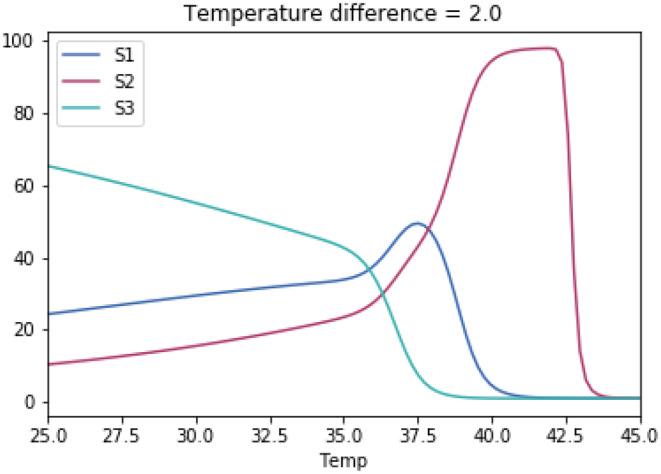
Competition of species across 25.0°C–45.0°C with differences in the temperature optimum of 2.0°C.

**FIGURE A24:**
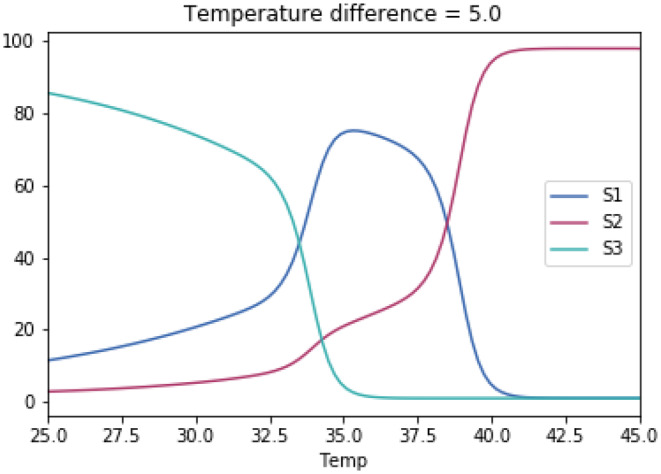
Competition of species across 25.0°C–45.0°C with differences in the temperature optimum of 5.0°C.

**FIGURE A25:**
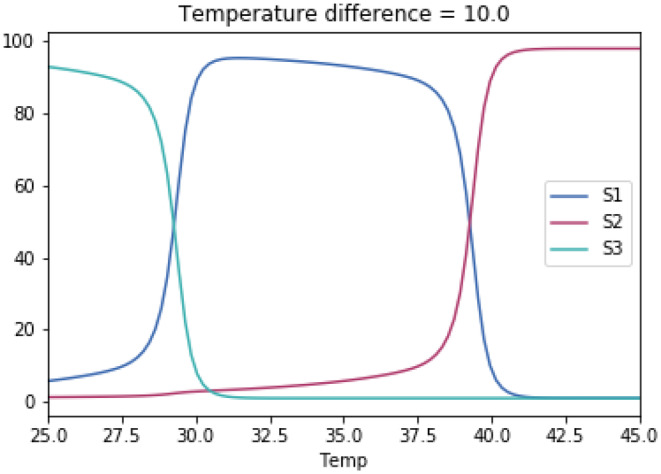
Competition of species across 25.0°C–45.0°C with differences in the temperature optimum of 10.0°C.

To determine how robust the model was to variations in number of competing species and temperature growth curves among competing species, we tested for the impacts of competing two versus three species by rerunning models across the tested temperature range for each pairwise combination of species (S_1_ vs. S_2_, S_1_ vs. S_3_, and S_2_ vs. S_3_). We then investigated the impact on model behavior of (1) normalization of the temperature–growth curves versus varying skewedness between species, (2) varying magnitude of skewedness in temperature–growth curves, and (3) varying the magnitude of the differences between the temperature optima of the curves (Figures A3, A4, A5, A6, A7, A8, A9, A10, A11, A12, A13, A14, A15, A16, A17, A18, A19, A20, A21, A22, A23, A24, A25). Our model results were highly consistent across these variations, except when the magnitude of variation among competing species in the skewedness or temperature optima of temperature growth curves were extremely small or large. It is worth noting, however, that the extent of variations we applied across these different scenarios are beyond what would be reasonably expected of thermal performance curves for typical contemporary organisms, further demonstrating the robustness of these findings. The code for the model setup as well as code testing the impact of various parameters is available at https://github.com/jen‐reeve/2024schipperetal.

## Author Contributions

**Louis A. Schipper:** conceptualization (lead), formal analysis (equal), funding acquisition (lead), investigation (lead), methodology (supporting), project administration (lead), resources (lead), supervision (equal), visualization (supporting), writing – original draft (lead), writing – review and editing (lead). **Jennifer L. Reeve:** conceptualization (supporting), data curation (equal), formal analysis (equal), investigation (equal), methodology (equal), visualization (equal), writing – review and editing (equal). **Vickery L. Arcus:** conceptualization (equal), funding acquisition (lead), investigation (supporting), project administration (equal), resources (equal), writing – review and editing (equal). **Terry Isson:** conceptualization (supporting), investigation (supporting), writing – original draft (supporting), writing – review and editing (supporting). **Erica J. Prentice:** writing – original draft (supporting), writing – review and editing (supporting). **Adele Williamson:** writing – original draft (supporting), writing – review and editing (supporting). **Andrew D. Barnes:** conceptualization (lead), formal analysis (lead), investigation (equal), methodology (equal), supervision (lead), visualization (lead), writing – original draft (equal), writing – review and editing (equal).

## Conflicts of Interest

The authors declare no conflicts of interest.

## Data Availability

The code for the model setup as well as code testing the impact of various parameters is available at https://github.com/jen‐reeve/2024schipperetal.

## Appendix A

### A.1 Model setup

The growth rate–temperature relationships of the three species had both different optima as well as different skewedness levels. The code for the model setup as well as code testing the impact of various parameters is available at https://github.com/jen‐reeve/2024schipperetal.

The relationships for each species were normalized to have the same maximum growth rate at each species' temperature optimum.

**TABLE A1:** Parameters used for generating the growth rate–temperature relationships used for Figure 1.3.

Species	Temperature optimum (°C)	Skewedness
S1	37.1	−10
S2	39.9	−15
S3	33.8	−5

For each temperature tested, the competition model was run to steady state, as shown at 37°C in Figure A2 in example.

### A.2 Model stress testing

#### A.2.1. 2 Species Competition

Competition models using the same growth rate temperature dependence but only two species at a time.

#### A.2.2. Impact of Normalizing the Growth Rates

Running the competition model with growth rate–temperature relationships that are identical to those used in Figure 1.3 except that they are not normalized to have the same growth rate at the temperature optimum.

#### A.2.3. Impact of Equal Skewedness of Growth Rate Temperature Relationships

Running the competition model with growth rate‐temperature relationships that are identical to those used in Figure 1.3 except that they have identical skewedness (skew = −10).

### A.3 Impact of Magnitude of Skewedness of Growth Rate Temperature Relationships

As with the previous test, the skewedness for all three species is the same in each test.

#### A.3.1. Impact of Varying the Differences in Temperature Between the Three Species

As with the previous tests, the skewedness for all three species is the same in each test. The temperature optimum for S1 in each test is 37.1°C. The temperature optimum for S2 for each test is 37.1°C + the temperature difference. The temperature optimum for S3 for each test is 37.1°C—the temperature difference.
